# Exploring the evolution of mass density and thickness of N-doped Ge-rich GeSbTe during multistep crystallization

**DOI:** 10.1038/s41598-024-65828-1

**Published:** 2024-06-25

**Authors:** Jacopo Remondina, Alain Portavoce, Yannick Le Friec, Daniel Benoit, Elisa Petroni, Magali Putero

**Affiliations:** 1 Aix Marseille Univ, CNRS, IM2NP, Marseille, France; 2 STMicroelectronics, 850 rue Jean Monnet, 38920 Crolles, France; 3 Smart PowerTechnology R&D, STMicroelectronics, Agrate Brianza, Italy

**Keywords:** Electronic devices, Phase transitions and critical phenomena, Phase transitions and critical phenomena

## Abstract

Among phase change materials, Ge-rich GeSbTe alloys (GGST) are key alloys for the next generation of embedded phase change memories because of their good thermal stability, allowing their use for the automotive applications. Several studies have investigated GGST crystallization, which takes place in several stages, including phase separation in the amorphous material, the crystallization of the cubic Ge and GST phases before a complete crystallization for higher thermal budget. So far, however, no information is available on the possible changes in density and thickness of such alloys. This paper investigates such variations in density and thickness for a N-doped GGST layer (GGSTN) during isothermal annealing, following the four main stages of its multistep crystallization process. X-ray reflectivity (XRR) and X-ray diffraction were employed for analysis. The study reveals that density and thickness exhibit distinct changes during crystallization, with density increasing by approximately 9% during transition from amorphous to crystalline states. These changes are attributed to alterations in layer morphology, particularly at the Ge crystallization temperature and at the onset of GST crystal formation. Additionally, at high thermal budgets, discrepancies between XRR analysis methods suggest the formation of a thin, lower density layer near the top interface of the GGSTN layer. These results provide insights into the structural evolution of the GGSTN layer, which is crucial for phase change random access memory applications.

## Introduction

Phase change materials (PCMs) are known to display markedly distinct optical and electrical properties, depending on their structural state, whether amorphous or crystalline^1,2^. As they can be reversibly switched very quickly between these two states via electrical pulses inducing Joule heating, they are key materials for phase change random access memories (PCRAM), which is the most mature and promising technology among emerging memories^3–6^. The most studied PCMs, namely the ternary Ge_2_Sb_2_Te_2_ (GST225) and binary GeTe alloys, have however a low crystallization temperature (150–170 °C for GST225 and 180–230 °C for GeTe)^3^. Consequently, both alloys cannot fulfil the desired data retention (2 years at 150 °C) for automotive applications and the desired stability at high temperature for soldering reflow compliance (typically 260 °C for 2 min)^6,7^. These drawbacks can be overcome by material engineering, by using Ge-rich GeSbTe alloys (GGST) exhibiting higher thermal stability, as initially shown by Cheng et al^8^. GGST alloys have actually been shown to have an higher crystallization temperature (> 300 °C)^9^ allowing enhanced devices performances^7,10,11^. Furthermore, the introduction of dopants such as N^12–14^, C^15^, As^16^ or O^17^ into Ge enriched GST has been proved to further enhance PCRAM performances, with a better stability of the amorphous phase, providing better contrast between the two states and very good electrical characteristics. In this study, N doping has been chosen. Several studies reported on the crystallization mechanism of GGST with and without N-doping, using sheet resistance, in and ex situ X-ray diffraction (XRD)^12,18–24^, Raman and Fourier Transform Infrared spectroscopies^25^, in and ex situ transmission electron microscopy (TEM) techniques^18,19,23,26,27^, X-ray photoelectron spectroscopy (XPS)^28^ and kinetic Monte Carlo simulations^22^. Several of these studies shown that the Ge-rich GST crystallization proceeds through the formation of small Ge grains first, followed by the formation of cubic stochiometric GST225 grains (see e.g.^19,20^). However, more recently, both GGST and N-doped GGST alloys have been shown to follow a much more complex and multistep crystallization mechanism^19,23,26^. In GGST, this mechanism involves, with increasing thermal budget^23,26^: (1) a phase separation in the amorphous phase, leading to Ge-rich and Ge-poor domains; (2) the nucleation of small crystals of Pnma GeTe that trigger the heterogeneous crystallization of Ge; (3) the crystallization of a cubic GST phase that is not the cubic GST225 but the cubic GeTe phase: at this stage, Ge and GeTe crystalline grains are still embedded in an amorphous Ge-rich matrix containing most of the Sb atoms; and (4) complete GGST crystallization that is obtained only by annealing the material above 400 °C, leading to the formation of cubic GST225 and some Sb-rich hexagonal phases. Adding N to GGST mainly changes the whole mechanism kinetics^12,14,20,29,30^: N tends to slow down the phase separation, crystallization, and growth processes during annealing, due to its interaction and bonding with Ge, that reduce the diffusivity of Ge in N-doped GGST.

For PCRAM applications, PCM mass density change upon crystallization/amorphization cycles is a key parameter: actually, as a common characteristic of PCMs, there is a significant volume reduction (6.5–9.6%)^31–33^ and a corresponding rise in mass density during crystallization. For the prototypical Ge_2_Sb_2_Te_5_ alloy, this results in substantial mechanical stresses within the PCM cells, leading to resistance drift and void formation in the device^34,35^. Ultimately, these factors may affect the cyclability of the memory cells, and some PCMs with zero mass density have been studied^36–38^. However, no data can be found about the mass density change of N-doped GGST material.

The present study focuses on the mass density and thickness changes upon the several steps of the crystallization mechanism of N-doped GGST (GGSTN) layers, characterized by x-ray reflectivity (XRR) and x-ray diffraction (XRD).

## Results and discussion

**Figure 1:**
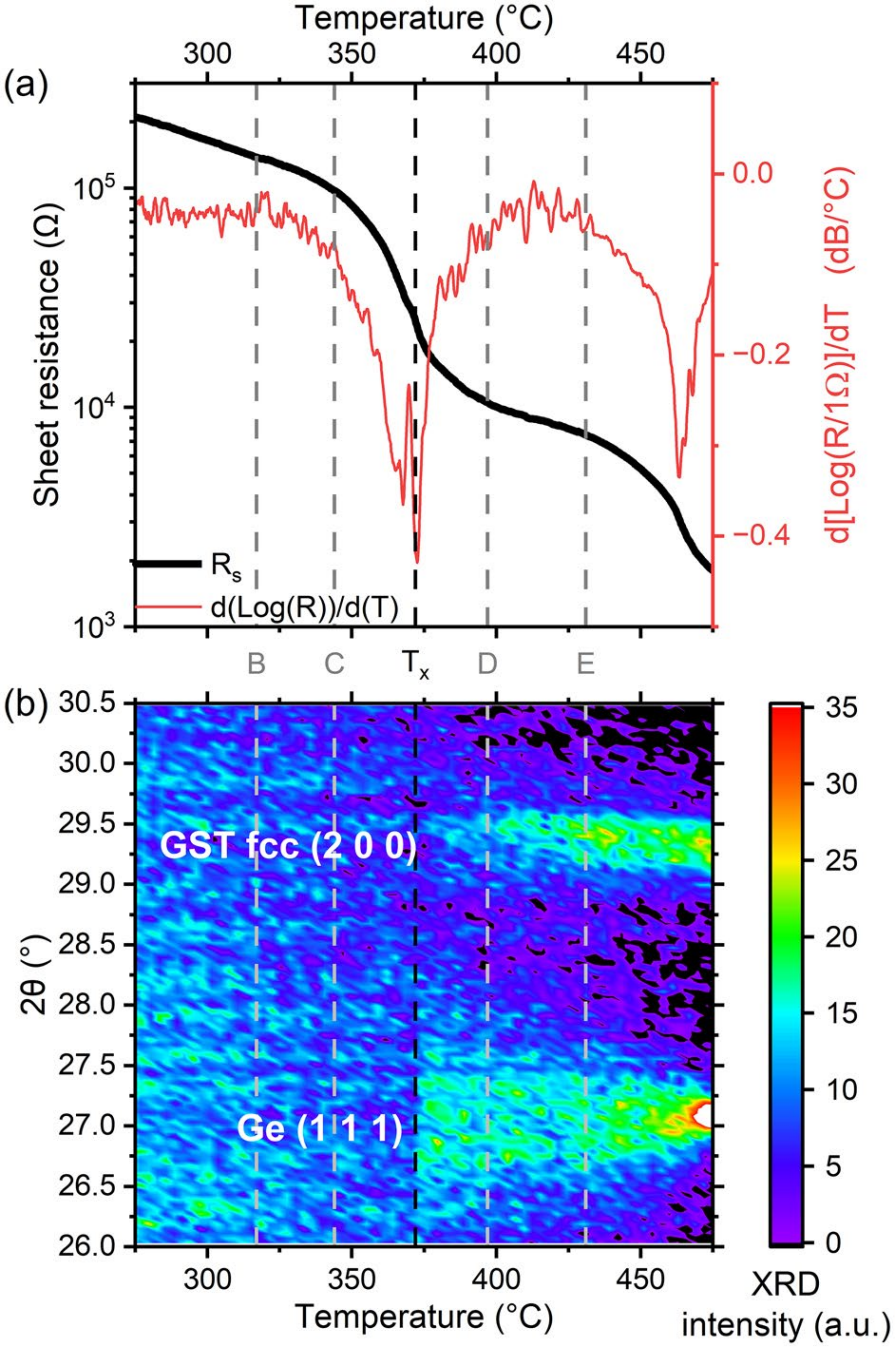
Combined in situ experiment (ramp 3 °C/min) used to determine the crystallization temperature Tx of the samples. (**a**) Sheet resistance (black line, left scale) and its derivative (red, right scale) after a local averaging filter. (**b**) Contour plot of the diffracted intensity (λ = 1.54 Å) after background subtraction. In both graphs, the vertical black line represents the temperature defined as “T_x_”, while the grey ones the temperatures chosen for isothermal annealing for samples B, C, D and E.

**Figure 2:**
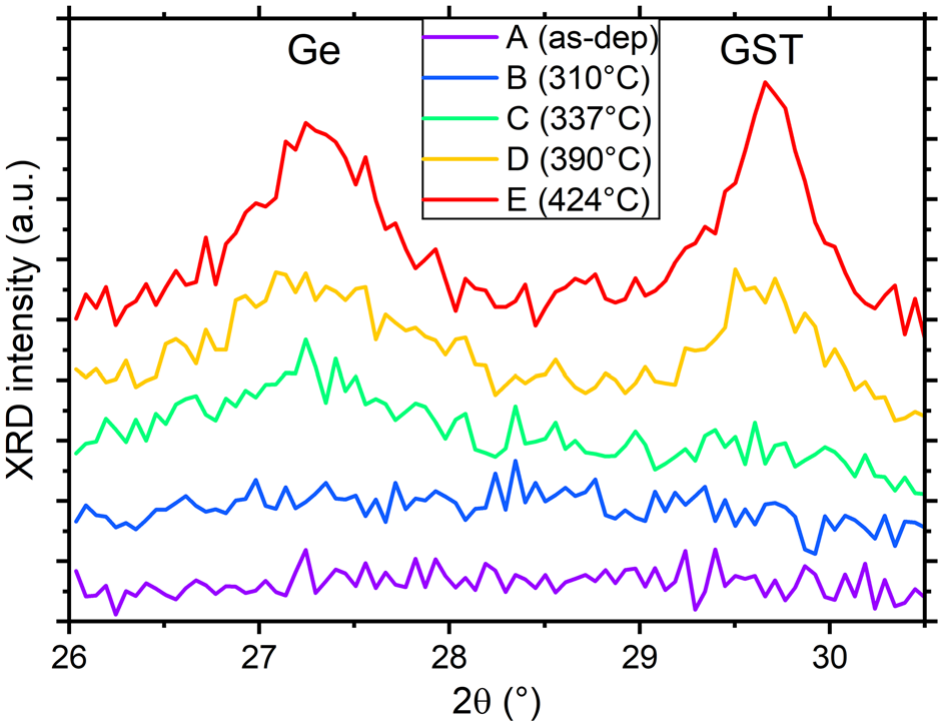
Part of XRD diffractograms (λ = 1.54 Å) after deposition (sample A) and after isothermal annealing (samples B, C, D and E) described in Table 1. The 2θ range is limited around the Ge (1 1 1) and GST fcc (2 0 0) peaks. The patterns have been shifted in intensity for clarity. The maximal annealing temperature is indicated for each sample.

A first slow ramp annealing (3 °C /min) was used to define the crystallization temperature of GGSTN (100 nm thick layer, capped with 20 nm SiN) using combined in situ XRD and sheet measurement (Rs) experiment. Figure 1 shows the combined XRD and Rs data, that were acquired during the same ramp annealing. The crystallization temperature (Tx) is defined as the temperature corresponding to the minimum of the Rs derivative, corresponding also to the appearance of Ge diffraction peak. Tx is found to be ~ 372 °C, which is in agreement with previous studies^14,30^. A second drop in Rs is clearly visible in Fig. 1a: it corresponds of an increase in both Ge and GST diffraction peaks (see Fig. 1b), indicating that the total crystalline fraction of the layer increases, leading to a decrease in sheet resistance. Following this first experiment, four samples were annealed at different temperatures and times, using isothermal annealing, and compared to the as-deposited sample (sample A). Table 1 summarizes the annealing conditions and expected crystalline state, corresponding to the four stages of the crystallization detailed in the introduction. Part of ex situ XRD patterns recorded on all the samples are shown in Fig. 2. For samples D and E, Rietveld refinements (see Fig. S1 in SI) were used to deduced the average relative fraction of Ge and GST phases, as well as their grain sizes, reported in Table 1. Figure 2 confirms that the layer is amorphous after deposition (sample A) and after the first isothermal annealing (sample B). However, phase separation has begun in sample B (annealing above 300 °C): the change of the shape of the background signal in XRD pattern, which depends on the short-range order inside the sample^39^, can indeed be due to phase separation [stage (1) of GGSTN crystallization]. Sample C, annealed at higher temperature but still below Tx, already shows a broad weak peak corresponding to the Ge (111) Bragg reflection^40^, but the GST (200) peak^41^ is still undetectable: this sample corresponds to the stage (2) of the GGSTN crystallization. For samples D and E, both Ge (111) and GST (200) Bragg reflections are present in the diffraction pattern, sample E exhibiting an increased intensity and area for both peaks compared to sample D. Rietveld refinements (see Table 1 and Fig. S1 in SI) and the average phase relative fractions confirm that the crystallization further increased between sample D and E: the relative phase fractions obtained in sample E for the Ge and the GST phases (resp. 63% and 37%) being almost that expected after full crystallization of the GGSTN layer. Thus, samples D and E respectively correspond to stages (3) and (4) of the GGSTN crystallization.

**Table 1:** Annealing conditions and crystallization state of the samples.

Sample name	A	B	C	D	E
Annealing	As-deposited	310 °C–10h	337 °C–4.5h	C + 390 °C–10 min	B + 424 °C–10 min
Crystallization step^1^		1	2	3	4
Crystalline state	Amorphous	Phase separation, amorphous	First Ge crystals	Ge + cubic GST crystals	Full crystallization
Rietveld refinement		FoM (χ^2^)		1.37	1.55
		Ge (%)		75%	63%
		GST (%)		25%	37%
		Ge grain size		5 nm	9 nm
		GST grain size		13 nm	13 nm

^1^For a description of the “crystallization steps” please refer to the introduction. For samples D and E, the main results from Rietveld refinements are given (relative phase fractions and average grain sizes, see supplementary information); for the phase fractions, the texturing and the presence of the GST hexagonal phase have been neglected: the fraction is as it appears comparing the Ge and the cubic GST peaks.

**Figure 3:**
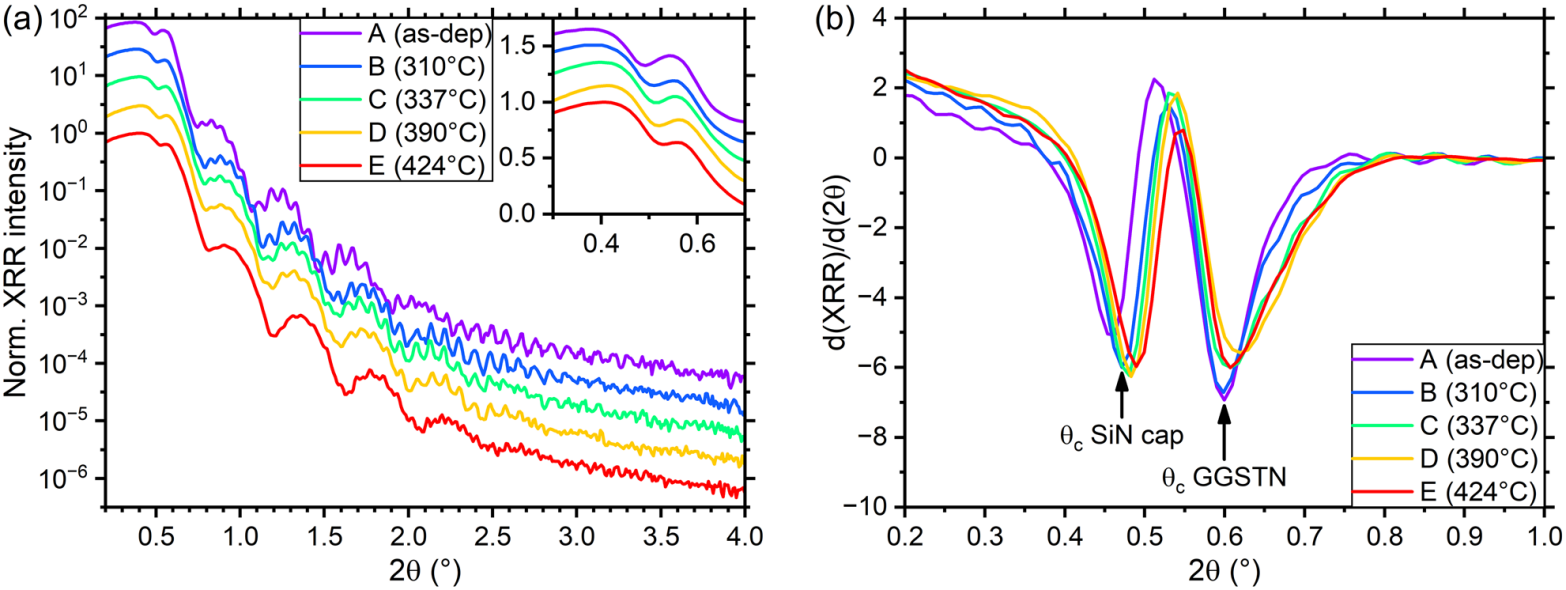
(**a**) XRR patterns (λ = 1.54 Å) for the investigated samples; data have been shifted in intensity for clarity. Inset: zoom around the critical angle region. (**b**) Analytical analysis of XRR patterns: derivatives of the curves highlighting the position of the critical angles (θc) for both SiN capping and GGSTN layers.

The XRR patterns recorded on all the samples are plotted in Fig. 3a. They show some differences after each annealing, reflecting the different crystallization stages. These differences affect not only the intensities of the narrow fringes (attributed to the GGSTN layer), that become less and less intense as the thermal budget increases, but also the broad fringes (related to the SiN capping layer). According to the XRR theory, the fading of the narrow fringes at higher thermal budget should be attributed to a fading of the electron density (ED) contrast between the layer associated to the fringes (GGSTN) and its neighbouring layers (the SiN capping layer and the thicker SiN underlayer), which could either correspond to (a) a change in the density of GGSTN, (b) a change in density of the SiN layers and/or (c) an increase of the interface roughness. In order to separate these effects, the variations in the measured critical angle(s) (θ_c_) must be considered, as they should only be affected by the layers’ density variations. Figure 3b presents the derivative of the XRR patterns and gives direct access to two critical angles (θ_c_), interpreted as the ones associated with the capping layer (at 2θ ~ 0.47°) and to the GGSTN layer (at 2θ ~ 0.6°). The results suggest that the densities of these two layers are indeed different for different annealing.

**Figure 4:**
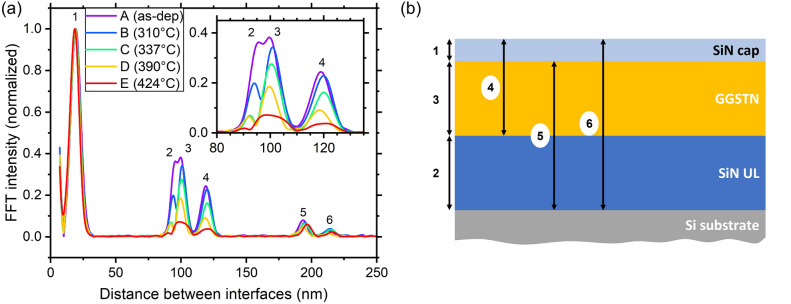
(**a**) Analytical analysis of XRR patterns: Fast Fourier Transform of the data in Fig. 3a so to extract layer thicknesses with (in the inset) a zoom on the region with the peaks corresponding to the underlayer (2), the GGSTN layer (3) and the GGSTN + capping combination (4). The intensities are normalized to the higher observed peak. (**b**) Diagram showing the sample stack and the different distances between interfaces corresponding to the FFT peaks labelled in (**a**).

The evolution of the fringes (broad and narrow) versus annealing was studied using the Fast Fourier Transform (FFT) of the XRR patterns^42^. Since the main focus of this paper concerns the GGSTN layer, the FFTs were calculated using the GGSTN critical angles (see Fig. 4a). All the FFTs patterns show six peaks. According to the intended stack (20 nm SiN/100 nm GGSTN/100 nm SiN/Si), the first peak (at ~ 20 nm) is attributed to the SiN capping layer, the fourth (~ 120 nm) to the sum of the capping layer and the GGSTN layer, the fifth (just below 200 nm) to the sum of the GGSTN and the SiN underlayer, and the sixth (~ 220 nm) to the total thickness of the stack (see Fig. 4b for FFT peaks and distance between interfaces correspondence). The second and third peaks at ~ 95 nm and ~ 100 nm could be both attributed either to the underlayer or the GGSTN layer, as they share the same nominal thickness (~ 100 nm). However, as the fourth peak at ~ 120 nm corresponds to the sum of the SiN cap and the GGSTN layer, and considering that the FFTs were normalized to the capping layer peak intensity, the variations of the peak at 120 nm should mainly reflect the variation of the GGSTN layer. Consequently, one would expect the peak corresponding to the GGSTN layer to vary in a similar way as the fourth peak at ~ 120 nm. One can see in Fig. 4a that the intensity and thickness variations of the peak at ~ 100 nm are similar to that of the peak at ~ 120 nm, while the peak at ~ 95 nm shows some differences (between annealing C and D for example). Thus, the second peak at ~ 95 nm is attributed to the SiN underlayer and the third peak at ~ 100 nm is attributed to the GGSTN layer.

According to these considerations, the data presented in the Figs. 3a and 4a give a qualitative feedback of the changes during the GGSTN crystallization process. The interference effects between all the interfaces are well defined in the XRR pattern of sample A, meaning that all the interfaces were very smooth before annealing (amorphous as deposited GGSTN). In sample B (amorphous annealed at 310 °C), the first interface to degrade is the one between the thick SiN underlayer and the substrate (roughness increase): this interface degradation is characterized by a significant decrease of the intensity of only the second FFT peak corresponding to the underlayer. The FFT of the XRR pattern of sample C shows an intensity decrease of the second, third, and fourth peaks, meaning that the interface between the SiN underlayer and the Si substrate gets probably even more degraded, but also the interface between the underlayer and the GGSTN. The FFT of the XRR pattern of sample D compared to that of sample C shows only a decrease of the intensity of the third (GGSTN layer) and fourth (GGSTN + SiN cap) peaks. Keeping in mind that the FFT intensities are normalized to that of the thin SiN cap, this means that only the interface between the thin SiN cap and the GGSTN layer is significantly degraded between annealing C and D. Finally, the FFT of the XRR pattern of sample E shows the simultaneous decrease of the peaks 2, 3, and 4, which should be mainly related to an increase of the roughness at least at both interfaces of the GGSTN layer. Besides, the positions of the FFT peaks shows that the SiN underlayer tends to become thinner (peak 2 at 95.5 nm for sample A compared to 90 nm for sample E) while the GGSTN layer first slightly increases in thickness (between samples A and B) but then shrinks again. Moreover, sample E clearly shows an asymmetry in the peak of GGSTN, such as if a second interface is appearing, towards higher thicknesses.

The electron and mass density of GGSTN layer as well as its thickness evolution were extracted from this analytical analysis by using the critical angle (θ_c_) position for mass density and the FFT data for layer thickness. The calculated values are reported in Table 2. The deduced values show that the main changes occur after the beginning of crystallization, with an increase in the average mass density. The thickness changes are however very small.

**Table 2:** Results from XRR analytical and simulation analysis, related to the GGSTN layer.

Sample name		A	B	C	D	E
Annealing		As-deposited	310 °C–10h	337 °C–4.5h	C + 390 °C–10 min	B + 424 °C–10 min
Crystallization step^1^		Amorphous	1 amorphous	2 (first Ge crystals)	3 (Ge + GST fcc)	4 (full crystallization)
Analytical^2^ GGSTN	θ_c_ (°)	0.297	0.296	0.303	0.309	0.301
	ρ_e_ (1/Å^3^)	1.26	1.25	1.31	1.37	1.30
	ρ_m_ (g/cm^3^)	4.99	4.98	5.19	5.39	5.09
	Thickness (nm)	100.2	101.1	100.6	99.9	–
Reflex^3^ GGSTN	Fit <|FoM|>	0.065	0.043	0.044	0.039	0.040
	Q_c_ (Å^−1^)	0.0420	0.0422	0.0427	0.0439	0.0440
	θ_c_ (°)	0.295	0.296	0.300	0.309	0.309
	ρ_e_ (1/Å^3^)	1.25	1.26	1.29	1.36	1.37
	ρ_m_ (g/cm^3^)	4.91	4.95	5.07	5.37	5.39
	Thickness (nm)	99.7	101.2	102.1	101.6	–

^1^For a description of the “crystallization steps” please refer to the introduction; ^2^ for the “analytical” analysis we measured the critical angle θ_c_, then calculated the scattering vector Q_c_ to calculate the densities; the estimated incertitude for the analytical measures are ± 0.005° for the critical angle (= scan step) and ± 2 nm for the thickness; ^3^Reflex uses the critical scattering vector Q_c_ to define the density of each layer and from them the electron density ρ_e_ and the mass density ρ_m_ can be calculated.

**Figure 5:**
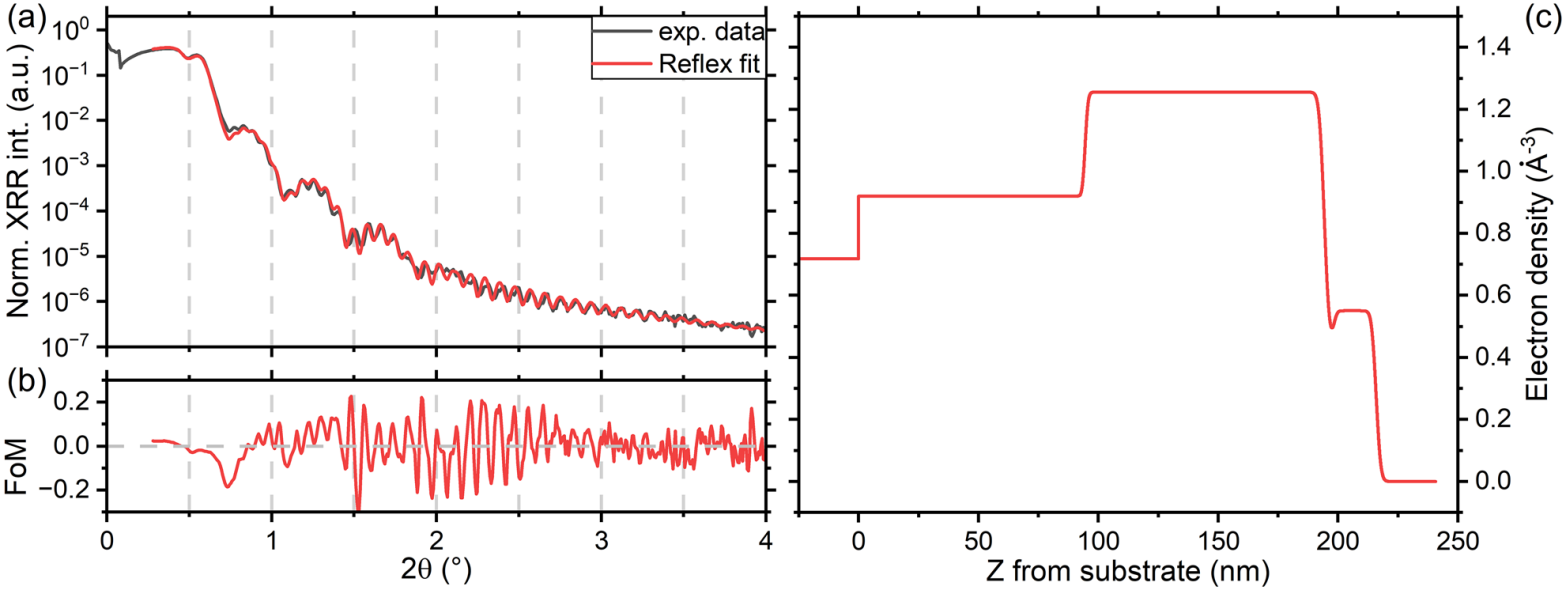
XRR simulations for the as-deposited sample (sample A): (**a**) XRR pattern from the experiment and the best simulation achieved from the simulation software; (**b**) FoM for the fits: log(I_fit_)–log(I_exp_); (**c**) the electron density (E.D.) profile corresponding to the simulated pattern. The equivalent figures for the other samples are in supplementary Figs. S2–S5.

XRR data were also simulated and fitted using a dedicated software^43^ enabling the optimization of density, thickness and roughness for each layer. However, the use of the sample nominal stack (i.e. 20 nm SiN (low density)/100 nm GGSTN/100 nm SiN/Si substrate) did not allow to correctly fit the XRR data, even for the amorphous sample: in particular, the region around the critical angles of the XRR patterns couldn’t be simulated properly, which is the most important for density evaluation of the GGSTN layer. Indeed, the critical angle regions in the XRR patterns mainly contain information up to the GGSTN layer, since the SiN underlayer should have an electron density lower than that of the GGSTN layer, preventing its critical angle to be probed. This simulation effect may be related to the fact that the software simulates interface roughness assuming that the density variations at the interface of two different layers follows an error function. To address this problem and properly simulate the XRR patterns, the capping layer was divided in two layers, with two different densities (higher at the surface, lower at the capping/GGSTN interface). This new layer (about 4 nm thick) can be seen either as an intermixing layer between GGSTN and SiN and/or as a way to simulate an interface roughness not following the error function. Dividing the capping layer into two layers allowed all the regions in the XRR patterns to be correctly simulated: Fig. 5 shows the results obtained for sample A, while the analogous figures for samples B to E can be found in the supplementary information Figs. S2–S5. All the simulations led to fits exhibiting good Figure of Merit (FoM) values (see Table 2), indicating a strong agreement between the model and the structure of the investigated samples. According to these fits (Fig. 5 and Figs. S2–S6 in supplementary information), the SiN cap/GGSTN interface is the most affected by thermal annealing, and plays the main role in the change of the observed XRR patterns. Actually, upon increasing thermal budget, this interface layer increases in thickness and roughness, starting to ~ 3.9 nm (+ 1 nm roughness) up to ~ 4 nm (+ 3 nm roughness) (see Figs. S2–S5). Its density also varies, with a clear increase as from sample D, just after Ge and GST fcc crystallization. Both the fits and the analytical analysis agree on the thicknesses, the densities for the GGSTN layer and, most importantly, on the evolution of these parameters versus annealing for all samples, excepted for sample E. This point will be discussed later. The results suggest that the total thickness of the capping layer (purple data in Fig. S7b in SI) decreases mainly after the first annealing (between samples A and B) and is almost constant up to sample E (slight decrease for the last annealing): the capping layer can thus sustain very well the annealing required for the operations of these materials.

**Figure 6:**
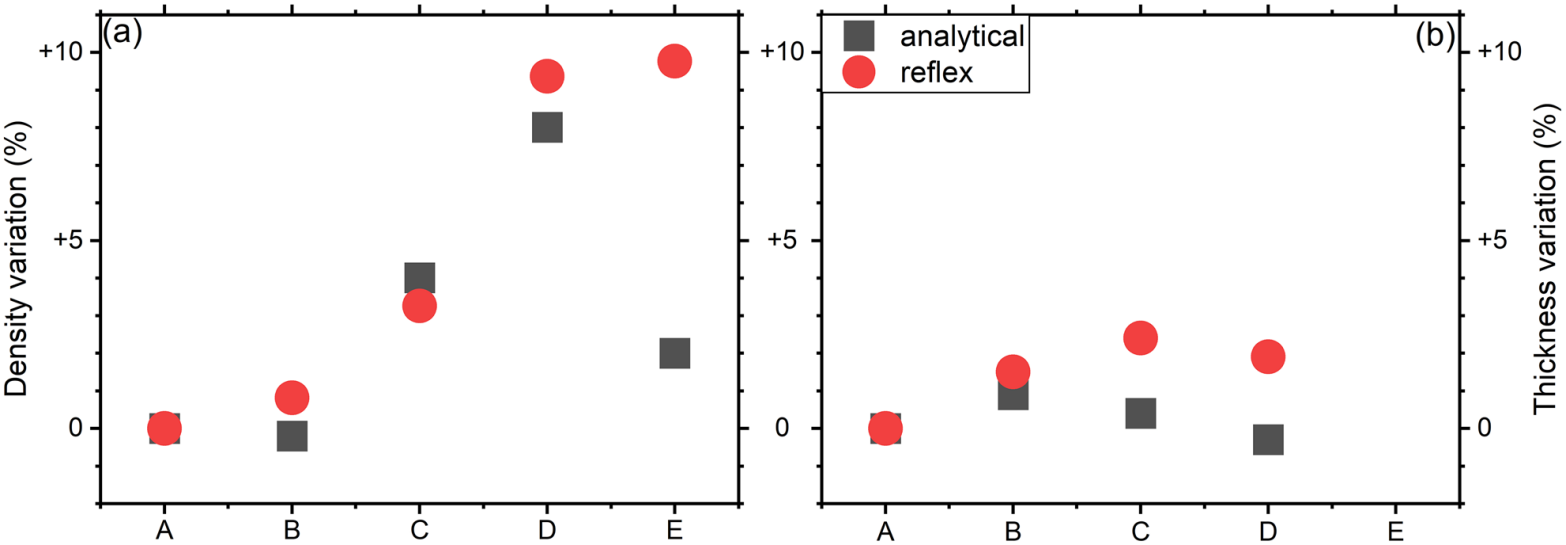
Density (**a**) and thickness (**b**) relative variations determined in the GGTN layer for the different samples submitted to an increasing thermal budget from samples A–E (see Table 1 for isothermal annealing description).

The GGSTN layer undergoes major changes as a consequence of the structural changes already described in the introduction. Part of these changes include a partial phase separation in regions richer in Ge (that later will form Ge-like crystals) and regions with a composition closer to that of the GST phase. Figure 6 summarises these changes for mass density and film thickness. After the last annealing the Kiessig fringes corresponding to the GGSTN layer are not clearly observed, due to an increase of the average roughness of several interfaces, especially the ones between GGSTN and the bottom and cap SiN layers (see SI Fig. S5): consequently, no reliable information on the thickness could be extracted, and for this reason they are not reported in Table 2 and Fig. 6b. The two analysis methods (analytical and simulation) generate very similar results and trends, excepted for the last annealing (sample E), probably due to the increased roughness of most interfaces. Table 2 and Fig. 6 show the general evolution of the GGSTN across the annealing (see also SI Figs. S6, S7): the layer density increases significantly after the second annealing step (sample C) and reaches a density increase up to ~ 9% once the film fully crystallized (sample E). However, the GGSTN layer thickness undergoes very little change, reaching a maximum increase of about 1–2%, depending on the analysis method. In general, the mass density and the thickness of a given film should evolve in opposite ways: a density decrease should correspond to a thickness increase, and vice versa. However, in our case, the results obtained using two different methods do not support this usual behaviour, as shown in Fig. 6a,b.

A first explanation for this unconventional behaviour could be linked to a high inhomogeneity of the layer not modelled in the fit of the XRR signal. Indeed, voids, amorphous (with different compositions) and crystallized regions (with several phases) have all been observed and documented to co-exist in GGSTN^19,26^. Despite the fact that, as a general rule, one can expect to measure an average electron density using XRR, these inclusions in the GGSTN layer possess closed interfaces that could lead to more complex interactions with reflected X-rays from flat interfaces, misleading XRR interpretations. A second possibility could be linked to N diffusion from the nitride layers into the GGSTN layer^21^. If N atoms are incorporated on interstitial sites in the crystalline grains in the GGSTN layer, the density of the layer could increase without significant change of its thickness. Third, one can note that the FFT analysis in the fully crystallized sample E (Fig. 4a) shows a peak 3 corresponding to the GGSTN layer with a bimodal shape. The fit of this peak using the convolution of two different Gaussian peaks gives two peaks separated with a distance of 4 nm. This distance could be the signature of the formation of a new layer of lower density between the SiN cap and the GGSTN layer, in agreement with the decrease of θ_c_ between sample D and E (Figs. 3b, 6a, black squares). The presence of this layer cannot be directly detected in the FFT due to its very small thickness (Fig. 4a). It is also important to mention that θ_c_ is particularly sensitive to surface effects, while simulations using the software Reflex average the layer density over its entire thickness, which could explain the discrepancy observed on density between the two methods in sample E (Fig. 6a, Table 2).

Nevertheless, it is important to stress that the extracted density variations match the prediction of the crystallization model described in the introduction:

1. During the phase separation (sample B), Ge-rich and Ge-poor regions are created, but they are still amorphous and consequently the layer density is not significantly modified in average, explaining why the GGSTN density in sample B is found to be similar to that in sample A. However, a slight increase of the GGSTN layer thickness of about 1.2% is observed (average of both methods), which could be explained by void formation already in the amorphous phase.
2. At the beginning of crystallization (sample C), small Ge crystals (diamond structure) appear, probably preceded by some GeTe transient phase crystals in the Pnma structure^23,26^. Schematically, this process can be seen as a phase separation, where GeTe-rich and Ge-rich regions are formed, while the amorphous matrix becomes enriched in Sb (which is the densest element), followed by the crystallization. At this stage, only small cubic Ge crystals are detected (Fig. 2), but we cannot excluded the presence of small GeTe Pnma crystals that trigger low-temperature Ge crystallization (337 °C, whereas Ge homogeneous crystallization is known to occur above 400 °C)^23^. Thus, the amorphous/crystal mass density changes concern mainly Ge and GeTe. Ge seems to keep its density constant^44,45^ between amorphous and crystalline phases, but it’s density (5.36 g/cm^3^) is higher than that of the amorphous GGSTN layer (~ 4.95 g/cm^3^, average value reported in Table 2). On the other hand, GeTe density increases for the formation of GeTe Pnma phase, which has a density of about 6.8 g/cm^3^ (no data are directly available about the density of this phase, but calculations starting from the Pnma cell^46^ with Ge and Te atoms at the predicted atomic positions, leads to a density of 6.8 g/cm^3^). Consequently, the density increase of ~  + 3.6% observed in sample C compared to that of the as-deposited sample is actually expected.
3. At the third stage of GGSTN crystallization (sample D), both cubic Ge and cubic GST (mainly cubic GeTe) crystalline grains are still embedded in an amorphous Ge-rich matrix containing most of the Sb atoms. As already mentioned, Ge should have a minor effect on the density variation, so the changes should mainly arise from the GeTe transformation. The Pnma/cubic transformation should lead to density decrease (from ~ 6.8 to ~ 6.16 g/cm^3^^47^), but due to the fact that the crystallized fraction increases, this effect could be counterbalanced, and an average increased of ~ 8.7% is measured for sample D, compared to the amorphous as-deposited sample.
4. Finally, at the last stage (full crystallization), lots of different phenomena are supposed to happen: the film can contain cubic-GeTe enriched in Sb exhibiting stoichiometries close to that of cubic-GST225, hexagonal phases rich in Sb, voids which may precipitate, etc.… These phenomena increase the layer complexity, which as a consequence hinder correct simulation of the XRR patterns and analytical analysis. This would explain why the analytical and the simulation/fit methods yield two different values for the (electron) density: the two methods respectively lead to a value of 1.30 e^−^/Å^3^ and 1.37 e^−^/Å^3^. The second value, extracted via simulation/fit, is very close to the value expected for pure crystalline Ge (i.e. 1.363 e^−^/Å^3^). Considering that the density of the GGSTN layer may not be uniform at this stage (i.e. the layer may be divided into two layers with two different densities), this value extracted from simulation/fit procedures should correspond to the thicker (main) part of the layer (Ge relative fraction of 63%, see Table 1), which is also the densest part of the GGSTN at this stage. Besides, as already mentioned, the first value of 1.30 e^−^/Å^3^ extracted with analytical method, and calculated from the critical angle position, should correspond to the upper part on the GGSTN layer. This value being lower, it leads to the conclusion that the GGSTN layer region near the upper interface should present a lower density, which could be explained with a higher concentration of voids/pores near the top SiN/GGSTN interface.

## Conclusions

In this work XRR and XRD measurements were used to study the variations of both density and thickness of a N-doped Ge-rich GST layer after sequential isothermal annealing involving a progressive thermal budget, corresponding to the main four stages of the multistep GGSTN crystallization process, starting from phase separation in the amorphous state up to full crystallization. XRR data were analysed using two different approaches, using either analytical method (critical angle measurement and FFT analysis), or using simulation/fit of the XRR patterns with a dedicated software. Although the determination of such parameters (density and thickness) in a multiphase and inhomogeneous layer remains a real challenge, our results give trends for each step of GGSTN crystallization process, GGSTN being an essential functional material for PCRAM memory applications. Both methods actually lead to the same trends for both density and thickness variations upon annealing, excepted for the highest thermal budget corresponding to full crystallization (above 400 °C). The global variation of the GGSTN layer thickness is not very pronounced up to 400 °C, and does not follow the expected behaviour compared to that of density, whatever the analysis method. This may be explained by (1) XRR theory and modelling, where unusual optical effects can appear due to the change in electron densities between the various amorphous, crystalline and void regions present in the GGSTN layer, and exhibiting various compositions, and/or by (2) intermixing layers formed at the GGSTN interfaces during the annealing. Nonetheless, our results show that the density of the GGSTN layer undergoes no major changes during the first crystallization stage characterized by phase separation at the amorphous state. However, the layer thickness slightly increases during this first stage, which could be linked to voids formation. During the transition from amorphous to crystalline, the GGSTN density tends to increase up to about 9%, which is common for PCM materials. These changes have been associated and explained thanks to a change in the morphology of the layer: the main density changes occur at the Ge crystallization temperature due to the crystallization process, as well as when the GST crystallization starts, just above this temperature. For the sample annealed at the highest thermal budget, the discrepancy between the two analysis (analytical and simulating/fitting methods) suggests that a new layer is formed at the top of the GGSTN layer, characterized by a very small thickness (~ 4 nm) and a lower mass density (in agreement with analytical data analysis), whereas the deeper part of the GGSTN layer keeps a similar density as during the previous crystallization stage (in agreement with the simulating/fitting analysis). These results provide insights into the structural evolution of the Ge-rich GST layer, crucial for PCRAM applications.

## Methods

100 nm thick GGSTN layers GGSTN (Ge > 40%at, N of few %at) were deposited by physical vapor deposition onto a 100 nm thick SiN layer, deposited on Si(100) wafer by Plasma Enhanced Chemical Vapour Deposition (PE-CVD). The GGSTN layer were capped by a 20 nm thick low density SiN layer. All the depositions were done without breaking the vacuum.

The samples were annealed in vacuum (*P* = 5 × 10^−5^ mbar) in a custom-made chamber that support the acquisition of both combined in situ XRD and sheet resistance measurements^48^, mounted on a Panalytical X’Pert diffractometer equipped with a Cu tube (λ = 1.54 Å) and an X’Celerator detector. Both ramp annealing (3 °C/min) and isothermal annealing were used, and the samples were let to cool down by thermalizing with the environment. Ex situ X-ray diffraction (XRD) and X-ray reflectometry (XRR) data have been acquired using a Panalytical Empyrean diffractometer equipped with a Cu tube (λ = 1.54 Å) and a PixCel 1D detector. Rietveld refinements were performed on the XRD patterns using the Profex software^49^, and the cubic Ge^40^ and GST phases^41^. Ex situ XRR patterns were simulated and fitted using Reflex software^43^.

### Supplementary Information

Supplementary Information.

## Data Availability

All data generated or analysed during this study are included in this published article (and its Supplementary Information files).
