# Supplementary material for: Exploring the evolution of mass density and thickness of N-doped Ge-rich GeSbTe during multistep crystallization
Source: Sci Rep. 2024 Jun 25;14:14677. doi: 10.1038/s41598-024-65828-1 (PMC11199619; doi:10.1038/s41598-024-65828-1)
Supplement: Supplementary file 1 — Supplementary Information. [file 41598_2024_65828_MOESM1_ESM.docx]

# Supplementary Information

Exploring the Evolution of Mass Density and Thickness of N-Doped Ge-Rich GeSbTe during Multistep Crystallization

Jacopo Remondina^1^, Alain Portavoce^1^, Yannick Le Friec^2^, Daniel Benoit^2^, Elisa Petroni^3^, and Magali Putero^1,*^

^1^ Aix Marseille Univ, CNRS, IM2NP, Marseille, France. *E-mail: [magali.putero@univ-amu.fr](mailto:magali.putero@univ-amu.fr)

^2^ STMicroelectronics, 850 rue Jean Monnet, 38920, Crolles, France.

^3^ Smart PowerTechnology R&D, STMicroelectronics, Agrate Brianza, Italy.


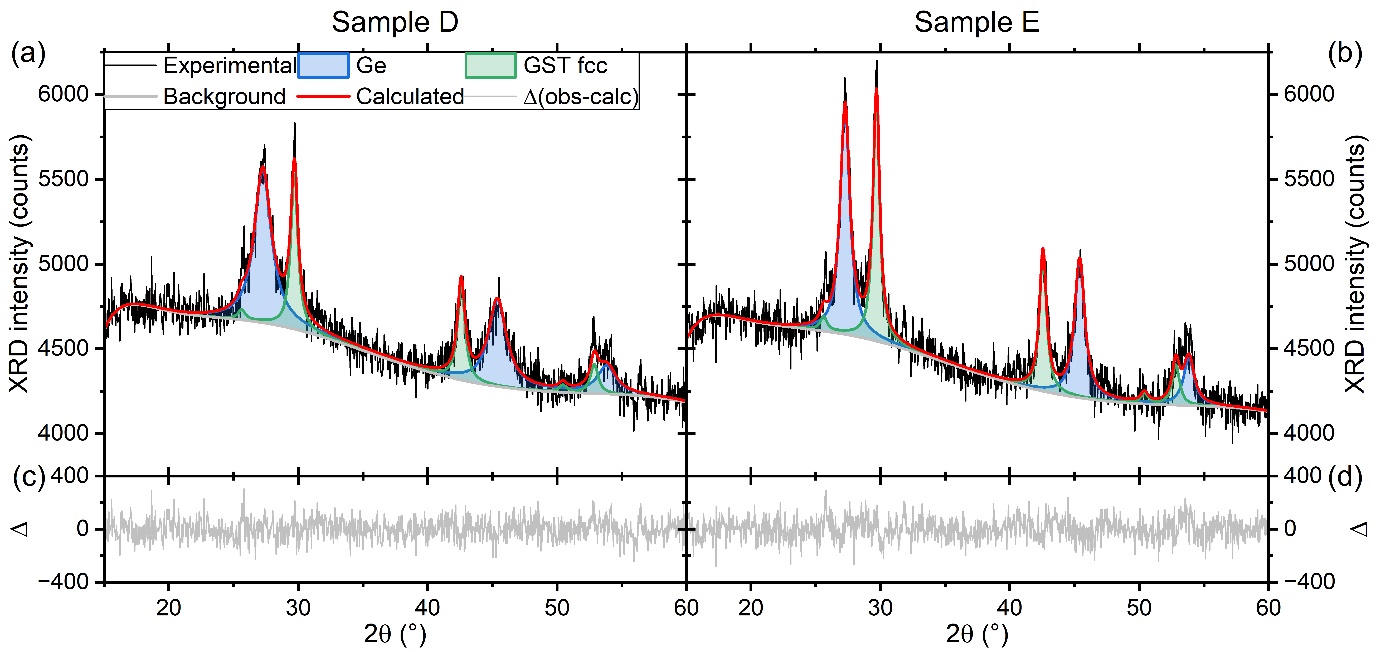


**Figure S1**: (a and b) Rietveld refinements for (a) sample D and (b) sample E with the experimental data, the complete refinement and the contributions from the background and each phase. (c and d) the residuals of the refinement defined as I_experimental_ - I_calculated_.

| **Sample name** | | **A** | | | |
| --- | --- | --- | --- | --- | --- |
| **Annealing** | | as-deposited | | | |
| **Crystallization step^1^** | | amorphous | | | |
| **Fit < \|FoM\| >** | | 0.065 | | | |
| **Reflex fit parameters** | **layer** | **SiN (sub)** | **GGSTN** | **SiN (cap)** | |
|  |  |  |  | bottom | top |
|  | **ρ_e_ (1/ Å^3^)** | 0.91 | 1.25 | 0.44 | 0.55 |
|  | **thickness (nm)** | 94.5 | 99.7 | 3.9 | 17.7 |
|  | **roughness (nm)** | 0.9 | 1.7 | 1 | 1.5 |

**Table S1**: Fitting parameters extracted from XRR simulations for sample A (see Fig.5 and Fig. S6).


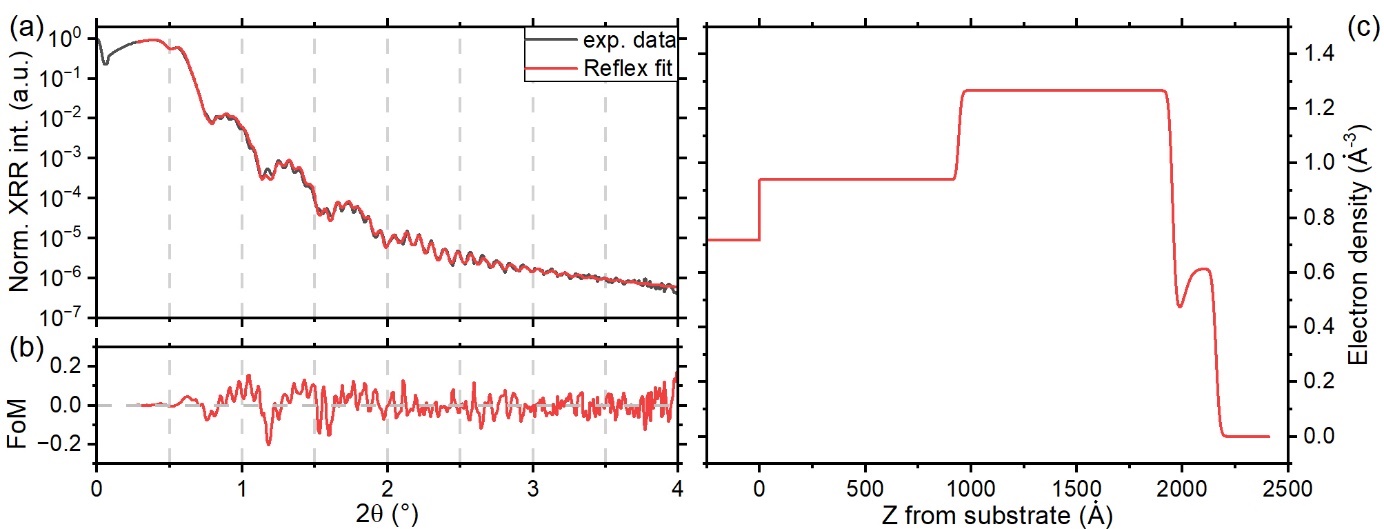


| **Sample name** | | **B** | | | |
| --- | --- | --- | --- | --- | --- |
| **Annealing** | | 310°C – 10h | | | |
| **Crystallization step^1^** | | i (amorphous) | | | |
| **Fit < \|FoM\| >** | | 0.043 | | | |
| **Reflex fit parameters** | **layer** | **SiN (sub)** | **GGSTN** | **SiN (cap)** | |
|  |  |  |  | bottom | top |
|  | **ρ_e_ (1/ Å^3^)** | 0.93 | 1.26 | 0.42 | 0.61 |
|  | **thickness (nm)** | 94.3 | 101.2 | 5.7 | 14.8 |
|  | **roughness (nm)** | 1.1 | 1.4 | 2.9 | 1.5 |

**Figure S2**: XRR simulations for sample B: (a) XRR pattern from the experiment and the best simulation achieved from the simulation software; (b) FoM for the fits: log(I_fit_)-log(I_exp_); (c) the E.D. profile corresponding to the simulated pattern. Bottom: Table with the fitting parameters extracted from simulation.


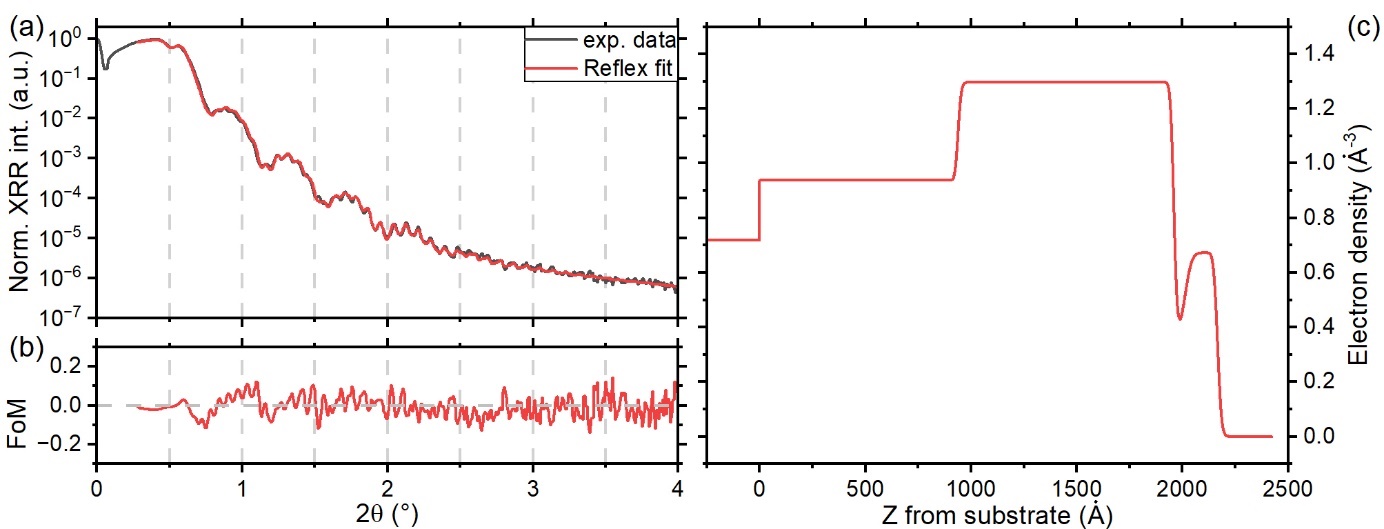


| **Sample name** | | **C** | | | |
| --- | --- | --- | --- | --- | --- |
| **Annealing** | | 337°C – 4.5h | | | |
| **Crystallization step^1^** | | ii (first Ge crystals) | | | |
| **Fit < \|FoM\| >** | | 0.044 | | | |
| **Reflex fit parameters** | **layer** | **SiN (sub)** | **GGSTN** | **SiN (cap)** | |
|  |  |  |  | bottom | top |
|  | **ρ_e_ (1/ Å^3^)** | 0.93 | 1.29 | 0.37 | 0.67 |
|  | **thickness (nm)** | 94.0 | 102.1 | 5.4 | 15.2 |
|  | **roughness (nm)** | 1.2 | 1.3 | 2.4 | 1.5 |

**Figure S3**: XRR simulations for sample C: (a) XRR pattern from the experiment and the best simulation achieved from the simulation software; (b) FoM for the fits: log(I_fit_)-log(I_exp_); (c) the E.D. profile corresponding to the simulated pattern. Bottom: Table with the fitting parameters extracted from simulation.


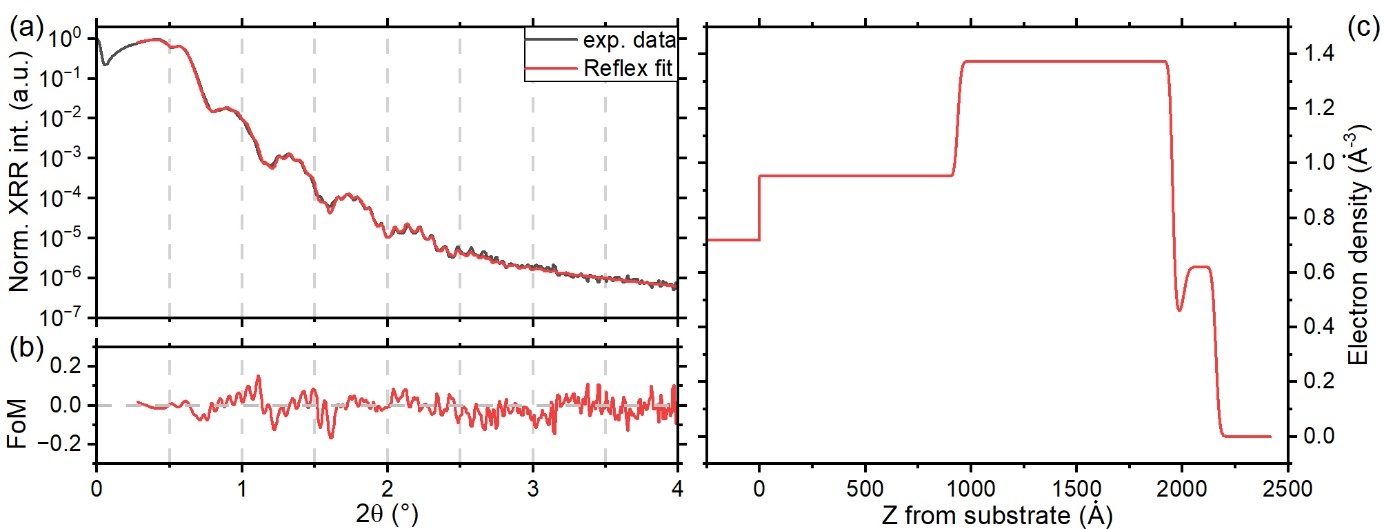


| **Sample name** | | **D** | | | |
| --- | --- | --- | --- | --- | --- |
| **Annealing** | | C + 390°C – 10 min | | | |
| **Crystallization step^1^** | | iii (Ge + GST fcc) | | | |
| **Fit < \|FoM\| >** | | 0.039 | | | |
| **Reflex fit parameters** | **layer** | **SiN (sub)** | **GGSTN** | **SiN (cap)** | |
|  |  |  |  | bottom | top |
|  | **ρ_e_ (1/ Å^3^)** | 0.95 | 1.36 | 0.44 | 0.62 |
|  | **thickness (nm)** | 93.8 | 101.6 | 5.5 | 14.9 |
|  | **roughness (nm)** | 1.3 | 1.2 | 1.6 | 1.4 |

**Figure S4**: XRR simulations for sample D: (a) XRR pattern from the experiment and the best simulation achieved from the simulation software; (b FoM for the fits: log(I_fit_)-log(I_exp_); (c) the E.D. profile corresponding to the simulated pattern. Bottom: Table with the fitting parameters extracted from simulation.


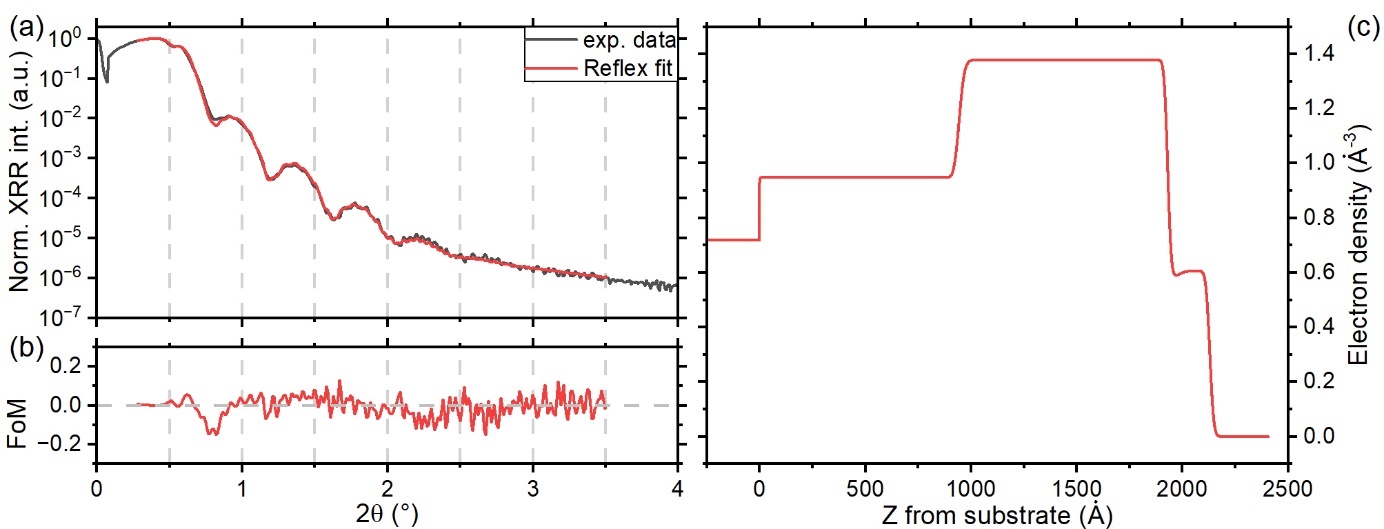


| **Sample name** | | **E** | | | |
| --- | --- | --- | --- | --- | --- |
| **Annealing** | | B + 424°C - 10 min | | | |
| **Crystallization step^1^** | | iv (full crystallization) | | | |
| **Fit < \|FoM\| >** | | 0.040 | | | |
| **Reflex fit parameters** | **layer** | **SiN (sub)** | **GGSTN** | **SiN (cap)** | |
|  |  |  |  | bottom | top |
|  | **ρ_e_ (1/ Å^3^)** | 0.94 | 1.37 | 0.56 | 0.6 |
|  | **thickness (nm)** | 94.4 | 98.6 | 4 | 16.9 |
|  | **roughness (nm)** | 1.9 | 1.4 | 3 | 1.5 |

**Figure S5**: XRR simulations for sample E: (a) XRR pattern from the experiment and the best simulation achieved from the simulation software; (b FoM for the fits: log(I_fit_)-log(I_exp_); (c) the E.D. profile corresponding to the simulated pattern. Bottom: Table with the fitting parameters extracted from simulation.


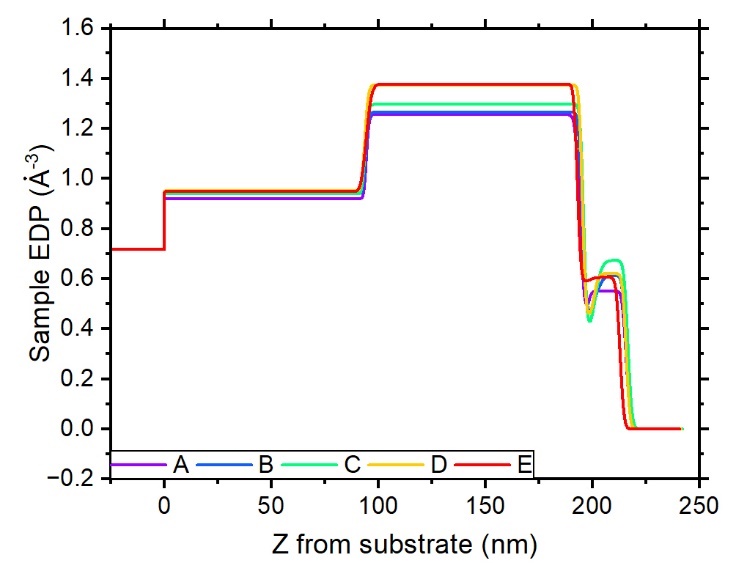


**Figure S6**: Electron densities (ED) profiles for all the investigated samples as resulted from simulations and fits using Reflex.


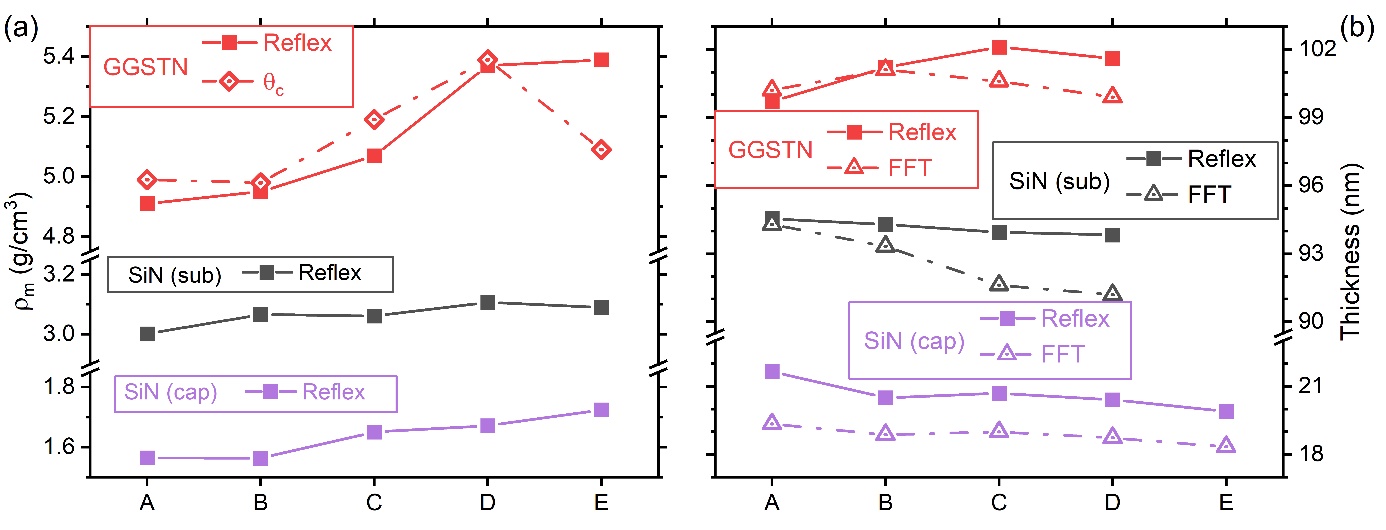


**Figure S7**: Evolution of the layers mass densities (a) and thicknesses (b) as a function of the annealing stage according to the different analysis methods used in this paper. SiN (sub) corresponds to the SiN underlayer, whereas SiN (cap) corresponds to the SiN capping layer, including an intermixing layer for SiN (cap).
